# Evaluation of a short training course of chest X-ray interpretation for the diagnosis of paediatric TB

**DOI:** 10.5588/ijtldopen.23.0484

**Published:** 2024-02-01

**Authors:** B. F. Melingui, E. Leroy-Terquem, M. Palmer, J-V. Taguebue, A. P. Wachinou, J. Gaudelus, A. Salomao, D. Bunnet, T. C. Eap, L. Borand, C. Chabala, C. Khosa, R. Moh, J. Mwanga-Amumpere, M. T. Eang, I. Manhiça, A. Mustapha, S. Beneteau, L. Falzon, J. A. Seddon, L. Berteloot, E. Wobudeya, O. Marcy, M. Bonnet, P. Y. Norval

**Affiliations:** ^1^ Recherches Translationnelles sur le VIH et les Maladies Infectieuses, University of Montpellier, Institut de Recherche pour le Développement (IRD), Institut national de la santé et de la recherche médicale (INSERM) Unité 1175, Montpellier,; ^2^ International Pulmonology Support, François Quesnay Hospital, Mantes-la-Jolie, France;; ^3^ Desmond Tutu TB Centre, Department of Paediatrics and Child Health, Stellenbosch University, Cape Town, South Africa;; ^4^ Mother and Child Center, Chantal Biya Foundation, Yaoundé, Cameroon;; ^5^ National Teaching Hospital for Tuberculosis and Pulmonary Diseases, Akpakpa Abokicodji, Cotonou, Benin;; ^6^ Service de Pediatrie, Hôpital Jean Verdier, Hôpitaux Universitaires Paris Seine Saint Denis, Bondy, France;; ^7^ Independent Public Health Consultant, Maputo, Mozambique;; ^8^ Epidemiology and Public Health Unit, Pasteur Institute in Cambodia, Phnom Penh,; ^9^ National TB Programme, Phnom Penh,; ^10^ Epidemiology and Public Health Unit, Clinical Research Group, Institut Pasteur du Cambodge, Phnom Penh, Cambodia;; ^11^ Center for Tuberculosis Research, Division of Infectious Diseases, Johns Hopkins University School of Medicine, Baltimore, MD, USA;; ^12^ Children’s Hospital, University Teaching Hospital, Lusaka, Zambia;; ^13^ Instituto Nacional de Saúde, Marracuene, Mozambique;; ^14^ Programme ANRS Coopération Côte d'Ivoire, Centre hospitalière universitaire de Treichville, Abidjan, Côte d’Ivoire;; ^15^ Epicentre Mbarara Research Centre, Mbarara, Uganda;; ^16^ National Center for Tuberculosis and Leprosy (CENAT/NTP), Ministry of Health, Phnom Penh, Cambodia;; ^17^ Programa Nacional de Controlo da Tuberculose, Ministério da Saúde, Maputo, Mozambique;; ^18^ Ola During Children Hospital, Freetown, Sierra Leone;; ^19^ International Pulmonology Support, Paris, France;; ^20^ Department of Infectious Disease, Imperial College London, London, UK;; ^21^ Department of Pediatric Radiology, University Hospital Necker-Enfants Malades, Assistance Publique – Hôpitaux de Paris, Paris, France;; ^22^ Makerere University-Johns Hopkins University Research Collaboration Care Ltd, Kampala, Uganda;; ^23^ University of Bordeaux, INSERM Unité mixte de Recherche 1219, IRD Unité Mixte de Recherche 271, Bordeaux,; ^24^ Technical Assistance for Management, Paris, France

**Keywords:** tuberculosis, diagnosis, children, chest X-ray, limited-resources countries

## Abstract

**BACKGROUND:**

Chest X-ray (CXR) interpretation is challenging for the diagnosis of paediatric TB. We assessed the performance of a three half-day CXR training module for healthcare workers (HCWs) at low healthcare levels in six high TB incidence countries.

**METHODS:**

Within the TB-Speed Decentralization Study, we developed a three half-day training course to identify normal CXR, CXR of good quality and identify six TB-suggestive features. We performed a pre–post training assessment on a pre-defined set of 20 CXR readings. We compared the proportion of correctly interpreted CXRs and the median reading score before and after the training using the McNemar test and a linear mixed model.

**RESULTS:**

Of 191 HCWs, 43 (23%) were physicians, 103 (54%) nurses, 18 (9.4%) radiology technicians and 12 (6.3%) other professionals. Of 2,840 CXRs with both assessment, respectively 1,843 (64.9%) and 2,277 (80.2%) were correctly interpreted during pre-training and post-training (*P* < 0.001). The median reading score improved significantly from 13/20 to 16/20 after the training, after adjusting by country, facility and profession (adjusted β = 3.31, 95% CI 2.44–4.47).

**CONCLUSION:**

Despite some limitations of the course assessment that did not include abnormal non-TB suggestive CXR, study findings suggest that a short CXR training course could improve HCWs’ interpretation skills in diagnosing paediatric TB.

## RÉSUMÉ

**CONTEXTE:**

L'interprétation de la radiographie thoracique (CXR) est un défi pour le diagnostic de la TB pédiatrique. Nous avons évalué la performance d'un module de formation de trois demi-journées sur la CXR destiné aux agents de santé (HCWs) dans six pays où l'incidence de la TB est élevée et où les ressources en services de santé sont limitées.

**MÉTHODES:**

Dans le cadre de l'étude de décentralisation TB-Speed, nous avons mis au point un cours de formation de trois demi-journées pour identifier une CXR normale, une CXR de bonne qualité et six caractéristiques suggestives de la TB. Nous avons effectué une évaluation avant et après la formation sur un ensemble prédéfini de 20 clichés radiologiques. Nous avons comparé la proportion de CXR correctement interprétées et le score médian de lecture avant et après la formation à l'aide du test de McNemar et d'un modèle linéaire mixte.

**RÉSULTATS:**

Sur les 191 HCWs, 43 (23%) étaient des médecins, 103 (54%) des infirmières, 18 (9,4%) des techniciens en radiologie et 12 (6,3%) d'autres professionnels. Sur 2 840 CXR avec les deux évaluations, respectivement 1 843 (64,9%) et 2 277 (80,2%) ont été correctement interprétées avant et après la formation (*P* < 0,001). Le score médian de lecture s'est amélioré de manière significative, passant de 13/20 à 16/20 après la formation, après ajustement par pays, établissement et profession (β ajusté = 3,31; IC 95% 2,44–4,47).

**CONCLUSION:**

Malgré certaines limites de l'évaluation du cours qui n'incluait pas de CXR anormale non évocatrice de TB, les résultats de l'étude suggèrent qu'une formation courte sur la CXR pourrait améliorer les compétences d'interprétation des HCWs dans le diagnostic de la TB pédiatrique.

The WHO estimates the incidence of childhood TB at 1.3 million cases per year, representing 12% of the global burden. However, only 49% of these cases were notified to the WHO by countries in 2022, and this low notification rate is assumed to be mainly due to underdiagnosis of TB.^1^ Childhood TB diagnostic challenges are generally due to the poor sensitivity of microbiological diagnostic tests in children, who generally have paucibacillary disease.^2^ In addition, microbiological tests are frequently underutilised because of the challenge of obtaining sputum.^3–5^ Consequently, most children with TB are not microbiologically diagnosed and their diagnosis relies on clinical evaluation and chest X-ray (CXR).^3^

Certain CXR features are particularly specific for intrathoracic TB, such as hilar lymph nodes, cavities and miliary TB but are poorly sensitive, and others like alveolar opacities and pleural effusion can be more sensitive but less specific.^6,7^ Furthermore, many studies have shown relatively low inter-reader reproducibility.^8,9^ In many high-incidence and resource-limited countries, clinicians lack CXR interpretation skills for the diagnosis of childhood intrathoracic TB. In addition to interpretation challenges, there is poor access to radiography in resource-limited countries, radiography being available at tertiary and secondary healthcare levels only; when available, such countries often face challenges with the maintenance of equipment, availability of supplies and poor quality of the images. CXR is rarely subsidised by national programmes.^9–11^

The WHO has recently recommended decentralising paediatric TB care to improve access to TB diagnosis and treatment in children.^12^ This requires strengthening diagnostic capacity at low healthcare levels, including access to good-quality CXR and training of CXR interpretation. As part of the TB Speed Decentralisation study, we developed and implemented a training course for healthcare workers (HCWs) from district hospitals (DHs) and primary health centres (PHCs) in CXR interpretation for the diagnosis of paediatric TB. Here, we present details of the implementation of the course and the outcomes of the pre–post-training assessments among HCWs at PHCs and DHs in six high TB incidence and resource-limited countries (Cambodia, Cameroon, Ivory Coast, Mozambique, Sierra Leone and Uganda).

## METHODS

### TB-Speed Decentralisation Study

This was an operational research study with a pre–post cross-sectional design to assess the effect of implementing a comprehensive childhood TB diagnostic package at the DH and PHC levels on paediatric TB case detection.^13^ The package included 1) symptom screening of all ill children below 15 years arriving at the health facility in order to identify children with presumptive TB, and in those identified with presumptive TB, 2) collecting samples for Xpert^®^ MTB/RIF Ultra (Cepheid; Sunnyvale, CA, USA) testing from nasopharyngeal aspirate and stool or sputum sample, 3) conducting clinical assessments, and 4) performing chest X-ray (CXR). Two districts were selected in each country with one DH and four PHCs per district.

CXR services were strengthened at the DH level by 1) setting up a digitalised system using digital radiography (DR) plates to improve quality and facilitate transfer of images, 2) introducing a training module in CXR interpretation developed by the project, and 3) setting up a quality assurance system for CXR reading by national re-readers.

### CXR training module

Training was intended to reinforce capacity of HCWs in identifying CXRs of acceptable quality, normal CXRs and CXRs suggestive of TB. The training course was designed for nurses with and without previous experience in CXR reading.

The course duration was set to be three half-days to ease implementation. The first part focused on the assessment of CXR quality and the different aspects of a normal antero-posterior (AP), postero-anterior (PA) and lateral CXR using a systematic approach (Supplementary Figure S1). Specific attention was given to the differentiation between the normal image of the thymus in children before 2 years of age and mediastinal adenopathy, and to the reading of lateral CXR. The second part focused on the identification of six features suggestive of childhood TB, following consensus in the TB-Speed CXR working group: 1) hilar or mediastinal adenopathy; 2) alveolar opacity; 3) intrathoracic airway compression, including atelectasis; 4) cavitation; 5) pleural or pericardial effusion, and 6) miliary pattern. We defined a TB-suggestive CXR by the presence of at least one of the six features.

The training course was developed in English and translated into French, Khmer and Portuguese. Images used for the training were anonymised CXRs collected by International Support for Pulmonology (ISP; Paris France) members during field missions. The course was approved by international experts and reviewed by the WHO Global Tuberculosis Programme and piloted with HCWs in Uganda not involved in the TB-Speed project prior to implementation. The course is available on the TB-Speed project website (https://www.tb-speed.com/fr/resources/).

The training was provided by a team of five pulmonologists, a paediatric infectious disease specialist and a public health physician in two health districts per country between November 2019 and January 2021. Course participants included HCW staff involved in the diagnosis and treatment of paediatric TB at the DH and PHC levels. Transport and accommodation were covered by the study.

### Evaluation of the course

An evaluation was performed prior to the training (pre-training test) with 20 CXRs (either AP, PA or lateral) from children, including two unreadable CXRs due to poor quality, 3 normal CXRs and 15 CXRs with at least one of the six features suggestive of TB (Supplementary Table S1 and Supplementary Figure S2). CXRs were selected by ISP among their CXR repository. The CXRs were projected onto a screen and participants were given one minute to record their interpretation onto a paper questionnaire with three possible options: unreadable CXR; normal CXR; CXR suggestive of TB. There was no opportunity for the participants to correct their answer after the pre-training test. After the test, participants did not receive correction of their answers. CXR selected for the test were not used during the training. At the end of the course (post-training test), participants were asked to interpret the same CXR set displayed in the same sequence as during the pre-training test with the same amount of time and same possible options.

At the end of the course, trainees were asked to complete a self-assessment questionnaire to report on the usefulness of the course, the perceived level of confidence in reading CXRs, the adequacy of the course content to their needs, the required degree of simplification for the interpretation of a TB-suggestive CXR, and the appropriateness of course duration, and to propose suggestions for improvement.

### Statistical analysis

We analysed data collected by the trainers in a MS Excel (Microsoft, Redmond, WA, USA) database for participants who responded to both the pre and post-training test questionnaires. Participant characteristics included sex, profession, place of work, prior CXR reading experience, number of CXRs interpreted in the month before the training and their country. The proportion of correctly interpreted CXR during the pre- and post-training test were presented overall, per country, and by participant characteristic. Comparisons between the proportion of correctly interpreted CXRs before and after training were made using the McNemar test for matched data. We assessed the effect of the training course on the CXR reading scores (number of correctly interpreted CXR out of 20 CXRs) adjusted to the participant characteristics found to be significantly associated with the reading score using a random-effect linear mixed model. All covariates with *P* < 0.20 in the univariate analysis were included in the multivariate model; those with *P* < 0.05 were retained for the final model. The Akaike Information Criterion (AIC) was used to select the best model to consider. Data were analysed using R software v4.2.1 (R Foundation for Statistical Computing, Vienna, Austria).

### Ethics statement

National ethics committees of each implementing country approved the study. Course participants were not asked to provide written consent.

## RESULTS

Of 219 course participants, characteristics were available for 191 HCWs, as this information was not collected in Mozambique. Of these, 64 (35%) were female, 43 (23%) were physicians, 103 (54%) nurses, 18 (9.4%) radiology technicians and 12 (6.3%) had another profession (nursing assistant and midwives). One hundred HCWs (56%) were employed by PHCs and 91 (44%) by DHs; 45 (25%) had previous CXR reading experience and 23 (17.0%) had read CXR in the month prior to the training. Characteristics per country are presented in Supplementary Table S2.

Data on the evaluation of the course by participants were not available for Uganda and Ivory Coast and for only 15/28 (53%) participants in Mozambique. Of 125 participants who filled out the questionnaire, 123 (98.4%) found the course to be useful or very useful, 124 (99.2%) appreciated the use of six TB-suggestive features, 21 (16.8%) found the course difficult to assimilate and 112 (89.6%) found it too short (Table 1).

**Table 1.** Participants' evaluation of the training course in Cambodia, Cameroon, Mozambique and Sierra Leone.*

	Cambodia	Cameroon	Mozambique	Sierra Leone	Total
	(*n* = 34)	(*n* = 37)	(*n* = 15)	(*n* = 39)	(*n* = 125)
	*n* (%)	*n* (%)	*n* (%)	*n* (%)	*n* (%)
Usefulness of the course to interpret CXR					
Very useful	26 (76.5)	33 (89.2)	12 (80.0)	35 (89.8)	106 ( 84.8)
Useful	8 (23.5)	3 (8.1)	3 (20.0)	3 (7.7)	17 (13.6)
Somewhat useful	0	0	0	1 (2.5)	1 (0.8)
Not useful	0	1 (2.7)	0	0	1 (0.8)
Gain in confidence in the interpretation of CXR after the course					
Yes	24 (70.6)	Not informed	15 (100.0)	28 (71.8)	67 (76.1)^†^
No	1 (2.9)	Not informed	0	3 (7.7)	4 (4.6)^†^
Other	9 (26.5)	Not informed	0	8 (20.5)	17 (19.3)^†^
Participant opinion on the classification of the 6 signs					
Very useful	17 (50.0)	35 (94.6)	10 (66.7)	29 (74.4)	91 (72.8)
Useful	16 (47.1)	2 (5.4)	5 (33.3)	10 (25.6)	33 (26.4)
Somewhat useful	1 (2.9)	0	0	0	1 (0.8)
Not useful	0	0	0	0	0
Level of the course					
Right level	16 (47.0)	33 (89.2)	12 (80)	28 (71.8)	89 (71.2)
Easy	8 (23.5)	3 (8.1)	2 (13.2)	2 (5.1)	15 (12.0)
Hard	10 (29.5)	1 (2.7)	1 (6.7)	9 (23.1)	21 (16.8)
Suggested correct course duration, days					
1.5^‡^	3 (8.8)	5 (13.5)	2 (13.3)	8 (20.5)	18 (14.0)
2–3	20 (58.9)	22 (59.5)	12 (80.0)	18 (46.1)	72 (57.6)
>4	11 (32.3)	10 (27.0)	1 (6.7)	13 (33.4)	35 (28.0)

* Suggestions from participants: more discussion of normal CXR according to age of participants; more time on lung anatomy; simplified version of the course to be used as job aid; continuous training after the course.

^†^ N = 88.

^‡^ 3 half-days corresponded to 1.5 day.

CXR = chest X-ray.

**Figure 1.**
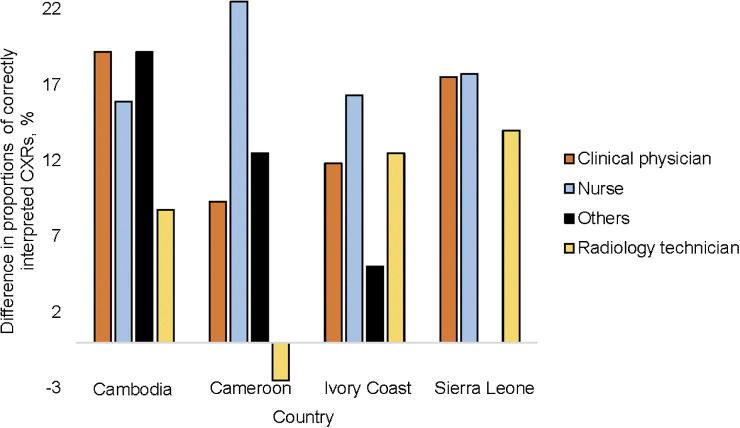
Difference between the proportions of chest X-ray correctly interpreted before and after training, by profession and by country.

Pre- and post-training evaluation data were available for all countries, except Uganda, resulting in 2,840 CXRs with both interpretations. Of these, 1,843 (64.9%) during pre-training and 2,277 (80.2%) during post-training readings were correctly interpreted (*P* < 0.001). The proportion of correctly interpreted CXR increased by 13% in physicians, 18% for nurses, 12% for people working in DHs, 16% for those working in PHCs and 16% in participants who had read CXRs in the month before the training (Table 2). The increase in the proportion of correctly interpreted CXRs by nurses ranged between 15% in Cambodia and 22% in Cameroon (Figure 1 and Supplementary Table S3). The highest proportion of correctly interpreted CXRs during the pre-training test was by radiographers across all countries (Supplementary Table S3).

**Table 2.** Results of the pre- and post-training assessment per participants socio-professional characteristics, level of healthcare facility and CXR reading experience.

Participant characteristics	CXR readings	Correctly interpreted CXR (pre-training)	Correctly interpreted CXR (post-training)	*P*-value*
	*n*	*n* (%)	*n* (%)	
Profession^†^				
Medical doctor	780	544 (69.7)	647 (82.9)	<0.001
Nurse	1,140	696 (61.1)	899 (78.9)	<0.001
Radiology technician	340	260 (76.5)	289 (85.0)	0.005
Other	180	106 (58.9)	135 (75.0)	0.001
Place of work^†^				
District hospital	1,160	825 (71.1)	968 (83.4)	<0.001
Primary health centre	1,360	823 (60.5)	1,059 (77.9)	<0.001
CXR interpreted in the month before training^†^				
Yes	460	356 (77.4)	397 (86.3)	<0.001
No	2,060	1,295 (62.9)	1,619 (78.6)	<0.001
Previous experience interpreting CXR				
Yes	880	627 (71.3)	741 (84.2)	<0.001
No	1,640	1,018 (62.1)	1,283 (78.2)	<0.001

* Mc Nemar's test.

^†^ Missing information: profession (n = 400 CXR readings); place of work (n = 320 CXR readings).

CXR = chest X-ray.

We observed a significant increase in the proportion of correctly interpreted CXRs after the training for all type of CXRs, except for those classified as unreadable due to poor inspiration and CXRs with alveolar opacities. This improvement was consistent across all professions, place of work and CXR reading experience (Supplementary Table S4). Normal CXRs with or without persisting thymus had a lower proportion of correct interpretations (14% and 39%, respectively) (Table 3). The median reading score improved significantly from 13/20 to 16/20 after training, after adjusting for the place of work, countries and profession (adjusted β = 3.31, 95% confidence interval 2.44–4.47) (Table 4).

**Table 3.** Results of pre- and post-training assessment per group of CXR with same patterns.

CXR patterns	CXR readings	Correctly interpreted CXR (pre-training)	Correctly interpreted CXR (post-training)	*P*-value*
	*n*	*n* (%)	*n* (%)	
Lymphadenopathies	426	286 (67.1)	357 (83.8)	<0.001
Lymphadenopathies + alveolar opacities	142	87 (61.3)	125 (88.0)	<0.001
Alveolar opacities	142	110 (77.5)	124 (87.3)	0.029
Alveolar opacities + lymphadenopathies + airways compression	142	123 (86.6)	132 (93.0)	0.078
Alveolar opacities + cavity within the alveolar opacity	142	127 (89.4)	137 (96.5)	0.020
Alveolar opacity + cavitation	142	125 (88.0)	137 (96.5)	0.008
Alveolar opacities	142	107 (75.4)	116 (81.7)	0.2
Atelectasis + lymphadenopathies	284	186 (65.5)	252 (88.7)	<0.001
Badly inspired film	142	55 (38.7)	59 (41.5)	0.6
Badly penetrated film	142	108 (76.1)	132 (93.0)	<0.001
Miliary	142	108 (76.1)	133 (93.7)	<0.001
Normal view with thymus	284	41 (14.4)	104 (36.6)	<0.001
Normal view without thymus	142	56 (39.4)	71 (50.0)	0.073
Opacities alveolar + lymphadenopathies	142	107 (75.4)	135 (95.1)	<0.001
Pericarditis	142	112 (78.9)	130 (91.5)	0.003
Pleurisy	142	105 (73.9)	133 (93.7)	<0.001

* Mc Nemar's test.

CXR = chest X-ray.

**Table 4.** Effect of the CXR training on the score of correctly interpreted CXR (*n* = 2,840 X-ray readings).

Characteristic	Course participants	Score	Bivariate analysis	*P*-value	Multivariate analysis	*P*-value
	*n*	Median [IQR]	β (95% CI)		Adjusted β (95% CI)	
Evaluation phase						
Pre-training	142	13.0 [11.0–15.0]	Reference		Reference	
Post-training	142	16.0 [15.0–18.0]	3.25 (2.41–4.38 )	<0.001*	3.31 (2.44–4.47)	<0.001
Place of work						
District hospital	58	16.0 [14.0–17.0]	Reference			
Primary health centre	68	14.0 [12.0–16.0]	0.56 (0.44–0.71)	<0.001*	0.65 (0.51–0.83)	<0.001*
Country						
Cambodia	34	15.0 [12.7–16.2]	Reference			
Sierra Leone	37	14.0 [12.0–16.0]	0.67 (0.46–0.98)	0.037*	0.57 (0.41–0.79)	<0.001*
Cameroon	32	15.0 [13.0–17.0]	1.15 (0.80–1.63)	0.449	0.82 (0.60–1.13)	0.231
Ivory Coast	39	16.0 [13.0–17.0]	1.15 (0.82–1.61)	0.416	1.09 (0.82–1.47)	0.550
Profession						
Physician	39	16.0 [14.0–17.0]	Reference		Reference	
Nurse	57	14.0 [13.0–16.0]	0.62 (0.48–0.80)	0.001*	0.69 (0.54–0.88)	0.003*
Radiology technician	17	16.0 [15.0–18.0]	1.44 (0.98–2.10)	0.062	1.38 (0.97–1.97)	0.075
Other	9	13.5 [11.0–16.0]	0.51 (0.33–0.81)	0.004*	0.52 (0.34–0.80)	0.003*
Previous experience interpreting CXR						
No	58	14.0 [12.0–16.0]	Reference			
Yes	58	16.0 [14.0–18.0]	1.72 (1.34–2.21)	<0.001*		

* Statistically significant.

CXR = chest X-ray; IQR = interquartile range; CI = confidence interval.

## DISCUSSION

Using simplified CXR interpretation principles, this three half-day training course increased the ability of HCWs from low healthcare level to correctly identify TB-suggestive CXRs, which could lead to improved diagnosis of paediatric TB in high-incidence and resource-limited countries. However, it remains to be seen whether this will have an impact on their clinical practice and improve their skills in the long term.

There was an improvement in CXR interpretation in all countries and across professions after the training, except in Cameroon in the case of radiology technicians. This is consistent with results from previous studies on training in CXR interpretation for TB diagnosis.^14–16^ In a study among adults with presumptive TB in New York, the median overall score for correct interpretation was 11 out of 20, with a significant correlation between score results and level of training.^14^ The use of a systematic reading approach using standardised methods can also improve skills to identify TB-suggestive radiological features in children.^17,18^

Nurses and participants from PHCs had more than 75% correct post-training test results, which suggests that CXR interpretation could be decentralised to PHC nurses. Radiology technicians had high reading scores prior to the training. They are used to reading CXRs to assess the quality of the images and could potentially be involved in CXR interpretation in settings with few doctors to avoid delays in results.

Regarding assessment of CXR quality, although most participants were able to identify good penetration, many had difficulties in ascertaining poor inspiration based on the number of posterior ribs visible.^19^ Participants had also challenges in identifying normal CXRs when there was no persisting thymus as such CXRs can be easily confused with mediastinal adenopathy. The limited ability to recognise normal CXRs is worrying, given that CXR, when available, is commonly used to rule out TB disease in child TB contacts prior to initiating TB preventive treatment (TPT). This may result in excluding children from TPT and, in the worst-case scenario, in overdiagnosing paediatric TB disease.^8^ Therefore, training in CXR interpretation for paediatric TB diagnosis should also focus on the identification of normal CXRs, particularly in young children. Highly specific radiological features, such as miliary patterns and cavities—less prevalent in children—were accurately recognised even in the pre-training test. Conversely, more prevalent features in children, like mediastinal lymphadenopathy, proved to be more challenging. The emphasis of the course was on using lateral CXR to identify hilar lymphadenopathy, particularly with the classical doughnut sign. This aspect appeared to be well understood, with over 80% of CXRs depicting mediastinal adenopathy being correctly interpreted in the post-training phase.^20^ However, lateral CXR is rarely done in routine practice due to the fear of radiation over-exposure and operational challenges, which may limit the reproducibility of the training findings without changes in practices.

The course was positively evaluated by most participants but many found the duration too short, and only 70% of participants in Cambodia and Sierra Leone felt confident about interpreting paediatric CXR after the course. Course duration was intentionally designed to be short to facilitate the implementation under routine conditions. The course provides basic knowledge of CXR reading for training-naïve HCWs and a good refresher for others, but is not adequate as a standalone tool and should be supplemented by mentoring by experienced clinicians, training in the use of a radiological atlas, quality assurance of CXR reading and additional refresher training courses.^7^

This study had several limitations. First, course participants were not asked to report which signs they identified on the CXR but only if the CXR was readable, normal or TB suggestive. We could not therefore assess the performance of the course in training HCWs to identify specific radiological features. However, we were able to retrospectively assess performance per group in identifying CXRs with the similar patterns.^11^ Second, the number of CXRs employed for both the pre- and post-training assessments was limited, particularly for certain features, leading to a reduced level of result precision. The CXRs were arranged in the same sequence for both the pre- and post-training evaluations, and the post-training assessment was conducted immediately after the conclusion of the course, both of which favoured the results of the post-training test. Third, the lack of additional assessments performed later is a major limitation of the assessment of the effect of the training, as the effect is expected to diminish with time. Fourth, as the set of CXRs used for the assessment comprised only unreadable, normal or TB-suggestive CXRs, excluding those that were abnormal but not suggestive of TB, there is a potential bias that might lead readers to over-interpret CXRs as TB-suggestive and consequently, overestimate accuracy. Finally, due to missing data from two countries (Uganda and Mozambique), our findings are not representative of the overall performance of the course in all TB-Speed study participating countries.

## CONCLUSION

Despite the limitations of the course, our findings suggest that a simplified short training module could improve the ability of HCWs from low healthcare levels to identify TB-suggestive CXRs. Wider dissemination of this course beyond the open access provided through the TB-Speed website is useful for programmatic use, but should be complemented with other existing training materials. Access to CXR remains a major bottleneck in many programmes; however, mobile portable X-ray machines are likely to lead to increased access. Further development of automated CXR reading, already validated in adults and currently under evaluation in children, could provide an alternative in high TB burden, resource-limited countries.^21^

## Supplementary Material


