# Supplementary material for: Evaluation of a short training course of chest X-ray interpretation for the diagnosis of paediatric TB
Source: IJTLD Open. 2024 Feb 1;1(2):76–82. doi: 10.5588/ijtldopen.23.0484 (PMC11221594; doi:10.5588/ijtldopen.23.0484)
Supplement: Supplementary file 1 [file iutld_ijtld_open_23.0484_supplementarydata1.pdf]

©2024 The Union http://dx.doi.org/10.5588/ijtldopen.23.0484

Evaluation of a short training course of chest X-ray interpretation for the diagnosis of paediatric TB

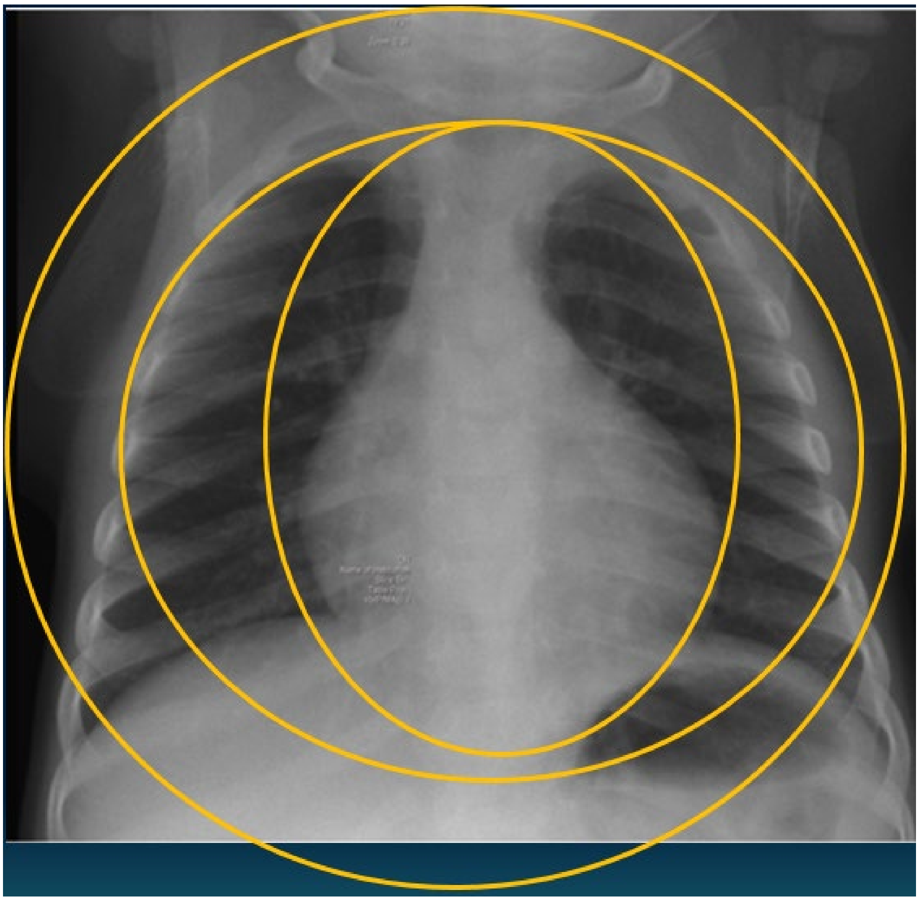

Supplementary Figure S1: Systematic approach with the three-circle method

<u>Supplementary Table S1:</u> Description of the Chest X- Rays used for the pre- and post-training assessments

Training slides	CXR features	CXR Classification according TB Speed training Module
**Slide 1 (AP and lateral CXR)**	Lymphadenopathies	Abnormal; suggestive of TB
**Slide 2 (AP and lateral CXR)**	Normal CXR without thymus	Normal; Not TB
**Slide 3 (AP and lateral CXR)**	Lymphadenopathies	Abnormal; suggestive of TB
**Slide 4 (AP and lateral CXR)**	Alveolar Opacities	Abnormal; suggestive of TB
**Slide 5 (AP and lateral CXR)**	Alveolar Opacity + Cavitation	Abnormal; suggestive of TB
**Slide 6 (AP)**	Badly inspired film	Unreadable
**Slide 7 (AP and lateral CXR)**	Lymphadenopathies +Alveolar Opacities	Abnormal; suggestive of TB
**Slide 8 (AP and lateral CXR)**	Opacities alveolar + Lymphadenopathies	Abnormal; suggestive of TB
**Slide 9 (AP and lateral CXR)**	Alveolar opacities	Abnormal; suggestive of TB
**Slide 10 (AP and lateral CXR)**	Atelectasis + Lymphadenopathies	Abnormal; suggestive of TB
**Slide 11 (AP CXR)**	Pleurisy	Abnormal; suggestive of TB
**Slide 12 (AP and lateral CXR)**	Miliary	Abnormal; suggestive of TB
**Slide 13 (AP and lateral CXR)**	Atelectasis + Lymphadenopathies	Abnormal; suggestive of TB
**Slide 14 (AP and lateral CXR)**	Normal view with thymus	Normal, not TB
**Slide 15 (AP and lateral CXR)**	Lymphadenopathies	Abnormal; suggestive of TB
**Slide 16 (AP CXR)**	Badly penetrated film	Unreadable
**Slide 17 (AP CXR)**	Alveolar Opacities + cavity within the alveolar opacity	Abnormal; suggestive of TB
**Slide 18 (AP CXR)**	Alveolar Opacities + Lymphadenopathies + Airways compression	Abnormal; suggestive of TB
**Slide 19 (AP CXR)**	Pericarditis	Abnormal; suggestive of TB
**Slide 20 (AP CXR)**	Normal view with thymus	Normal; not TB

AP antero-posterior; PA : postero anterior; CXR: Chest – X- ray; TB :Tuberculosis

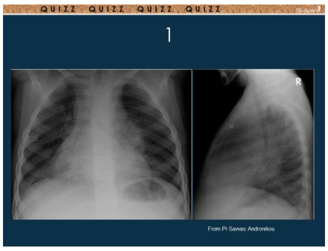
QUIZZ QUIZZ QUIZZ QUIZZ
1
From Pr Savvas Andronikou

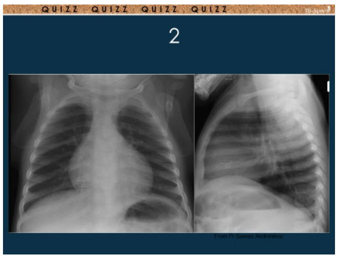
QUIZZ QUIZZ QUIZZ QUIZZ
2

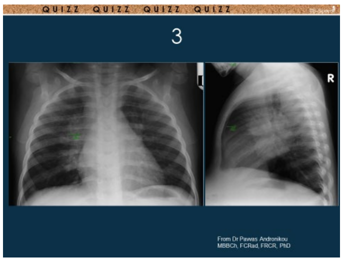
QUIZZ QUIZZ QUIZZ QUIZZ
3
From Dr Savvas Andronikou
MBBCh, FCRad, FRCR, PhD

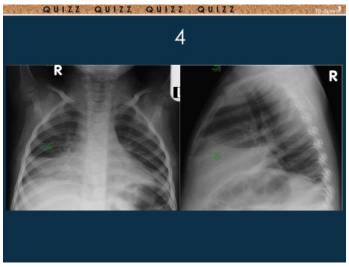
QUIZZ QUIZZ QUIZZ QUIZZ
4

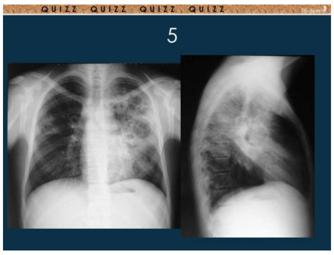
QUIZZ QUIZZ QUIZZ QUIZZ
5

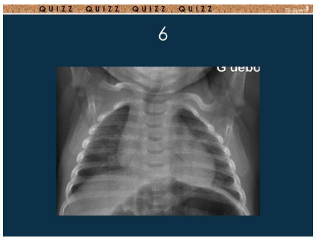
QUIZZ QUIZZ QUIZZ QUIZZ
6

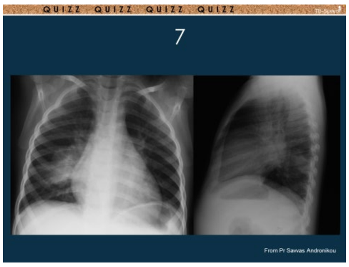
QUIZZ QUIZZ QUIZZ QUIZZ
7
From Pr Savvas Andronikou

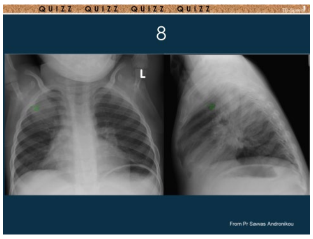
QUIZZ QUIZZ QUIZZ QUIZZ
8
From Pr Savvas Andronikou

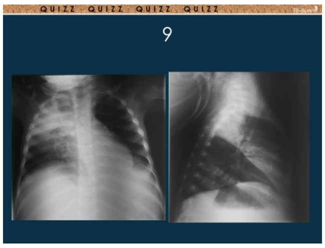
QUIZZ QUIZZ QUIZZ QUIZZ
9

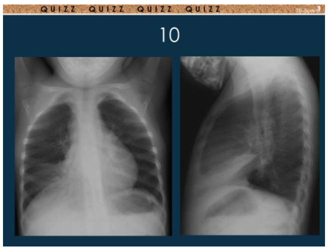
QUIZZ QUIZZ QUIZZ QUIZZ
10

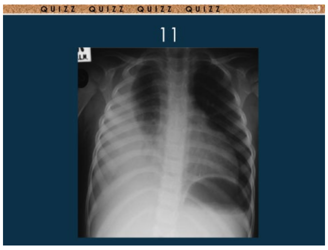
QUIZZ QUIZZ QUIZZ QUIZZ
11

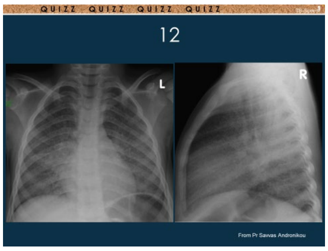
QUIZZ QUIZZ QUIZZ QUIZZ
12
From Pr Savvas Andronikou

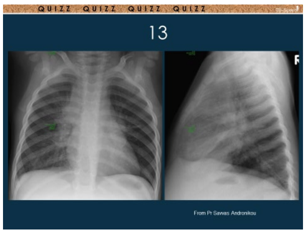
QUIZZ QUIZZ QUIZZ QUIZZ
13
From Pr Savvas Andronikou

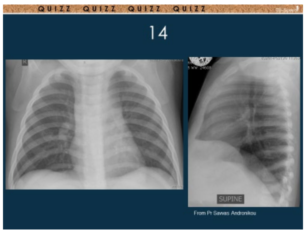
QUIZZ QUIZZ QUIZZ QUIZZ
14
SUPINE
From Pr Savvas Andronikou

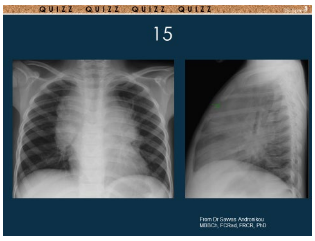
QUIZZ QUIZZ QUIZZ QUIZZ
15
From Dr Savvas Andronikou
MBBCh, FCRad, FRCR, PhD

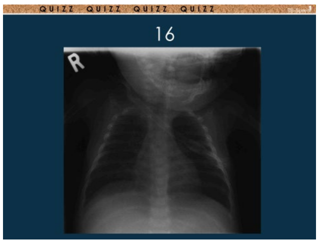
QUIZZ QUIZZ QUIZZ QUIZZ
16

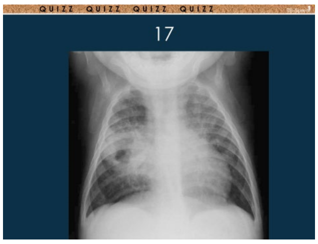
QUIZZ QUIZZ QUIZZ QUIZZ
17

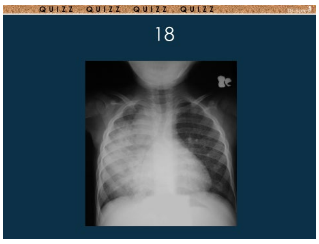
QUIZZ QUIZZ QUIZZ QUIZZ
18

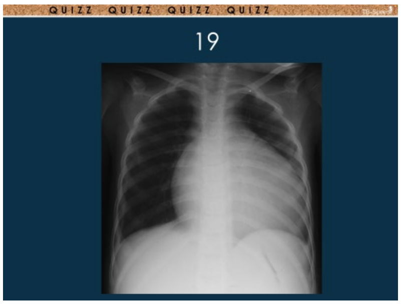

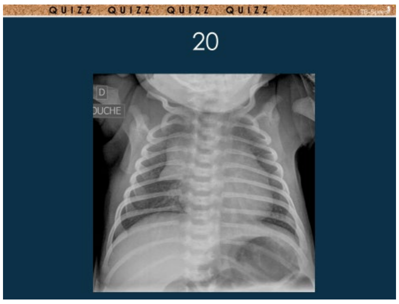

<u>Supplementary Figure S2</u>: Set of 20 chest X-rays used for the pre- and post-training assessments

Supplementary Table S2: Characteristics of the course participants by country

Characteristics	Cambodia, N = 34	Cameroon, N = 32	Ivory Coast, N = 39	Sierra Leone, N = 37	Uganda, N = 49	Overall, N = 191
Age, median (IQR)	40 (30, 49)	34 (30, 42)	37 (32, 44)	NA	37 (31, 42)	37 (31, 45)
Sex, n (%)						
Female	9 (26.5)	16 (50.0)	4 (10.3)	12 (40.0)	23 (47.9)	64 (35.0)
Male	25 (73.5)	16 (50.0)	35 (89.7)	18 (60.0)	25 (52.1)	119 (65.0)
Place of Work, n (%)						
District Hospital	10 (29.4)	25 (80.0)	14 (35.9)	10 (34.5)	19 (42.2)	78 (43.5)
Primary Heath Centre	24 (70.6)	6 (20.0)	25 (64.1)	19 (65.5)	26 (57.8)	100 (56.5)
Previous experience interpreting CXR, n (%)	9 (26.5)	16 (55.2)	11 (28.2)	8 (26.7)	1 (2.0)	45 (24.9)
CXR interpreted last month before training, n (%)	1 (2.9)	9 (31.0)	12 (30.8)	1 (2.8)	0 (0)	23 (16.7)
Position, n (%)						
Medical Doctor	6 (17.6)	14 (43.8)	11 (28.2)	8 (21.6)	4 (8.2)	43 (22.5)
Nurse	16 (47.1)	12 (37.5)	23 (59.0)	11 (29.7)	41 (83.7)	103 (53.9)
Radiology Technician	4 (11.8)	4 (12.5)	4 (10.3)	5 (13.5)	1 (2.0)	18 (9.4)
Others	7 (20.6)	2 (6.3)	1 (2.6)	0 (0.0)	2 (4.1)	12 (6.3)
Not specify	1 (2.9)	0 (0.0)	0 (0.0)	13 (35.1)	1 (2.0)	15 (7.9)

CXR: chest x-ray

Supplementary Table S3: Results of the pre- and post-training assessment by participant's profession and countries

	Medical Doctor			Nurse			Radiology Technician			Others			Not specify		
**Characteristic**	**Pre-training, N = 780**	**Post-training, N = 780**	**p-value[1]**	**Pre-training, N = 1,140**	**Post-training, N = 1,140**	**p-value[1]**	**Pre-training, N = 340**	**Post-training, N = 340**	**p-value[1]**	**Pre-training, N = 180**	**Post-training, N = 180**	**p-value[2]**	**Pre-training, N = 400**	**Post-training, N = 400**	**p-value[1]**
Cambodia, n/N (%)	85/120 (70.8)	108/120 (90.0)	<0.001	142/220 (64.5)	177/220 (80.5)	<0.001	60/80 (75.0)	67/80 (83.8)	0.2	65/120 (54.2)	88/120 (73.3)	0.002	72/140 (51.4)	106/140 (75.7)	<0.001
Cameroon, n/N (%)	211/280 (75.4)	237/280 (84.6)	0.006	136/240 (56.7)	190/240 (79.2)	<0.001	68/80 (85.0)	66/80 (82.5)	0.7	24/40 (60.0)	29/40 (72.5)	0.2	NA	NA	-
Ivory coast, n/N (%)	161/220 (73.2)	187/220 (85.0)	0.002	290/460 (63.0)	365/460 (79.3)	<0.001	63/80 (78.8)	73/80 (91.3)	0.027	17/20 (85.0)	18/20 (90.0)	>0.9	NA	NA	-
Sierra Leone, n/N (%)	87/160 (54.4)	115/160 (71.9)	0.001	128/220 (58.2)	167/220 (75.9)	<0.001	69/100 (69.0)	83/100 (83.0)	0.020	NA	NA		165/260 (63.5)	201/260 (77.3)	<0.001

[1]Mc Neymar test; NA: Not Applicable

Supplementary Table S4: Results of pre- and post-training reading assessment per group of chest X-rays and participants profession

	Medical Doctor			Nurse			Radiology Technician			Others			Not specify		
**Characteristics**	**Pre-training**,, N = 780	**Post-training**, N = 780	**p-value**[1]	**Pre-training**,, N = 1,140	**Post-training**, N = 1,140	**p-value**[1]	**Pre-training**,, N = 340	**Post-training**, N = 340	**p-value**[1]	**Pre-training**, N = 180	**Post-training**, N = 180	**p-value**[1]	**Pre-training**,N = 400	**Post-training**, N = 400	**p-value**[1]
Adenopathies, n(%)	84 (71.8)	95 (81.2)	0.090	103 (60.2)	145 (84.8)	<0.001	43 (84.3)	45 (88.2)	0.6	17 (63.0)	24 (88.9)	0.026	39 (65.0)	48 (80.0)	0.066
Adenopathies +Alveolar Opacities, n (%)	24 (61.5)	30 (76.9)	0.14	34 (59.6)	54 (94.7)	<0.001	14 (82.4)	15 (88.2)	>0.9	5 (55.6)	8 (88.9)	0.3	10 (50.0)	18 (90.0)	0.006
Alveolar Opacities, n(%)	34 (87.2)	38 (97.4)	0.2	39 (68.4)	48 (84.2)	0.047	14 (82.4)	15 (88.2)	>0.9	6 (66.7)	7 (77.8)	>0.9	17 (85.0)	16 (80.0)	>0.9
Alveolar Opacities + adenopathies + Airways compression, n (%)	35 (89.7)	38 (97.4)	0.4	48 (84.2)	54 (94.7)	0.067	17 (100.0)	17 (100.0)		7 (77.8)	5 (55.6)	0.6	16 (80.0)	18 (90.0)	0.7
Alveolar Opacities + cavity within the alveolar opacity, n (%)	37 (94.9)	38 (97.4)	>0.9	48 (84.2)	56 (98.2)	0.008	17 (100.0)	17 (100.0)		8 (88.9)	8 (88.9)	>0.9	17 (85.0)	18 (90.0)	>0.9
Alveolar Opacity + Cavitation, n (%)	37 (94.9)	38 (97.4)	>0.9	51 (89.5)	56 (98.2)	0.11	17 (100.0)	17 (100.0)		6 (66.7)	7 (77.8)	>0.9	14 (70.0)	19 (95.0)	0.091
Alveolar opacities, n(%)	32 (82.1)	35 (89.7)	0.3	38 (66.7)	45 (78.9)	0.14	15 (88.2)	15 (88.2)	>0.9	8 (88.9)	6 (66.7)	0.6	14 (70.0)	15 (75.0)	0.7
Atelectasis + adenopathies, n(%)	54 (69.2)	67 (85.9)	0.013	70 (61.4)	102 (89.5)	<0.001	26 (76.5)	28 (82.4)	0.5	12 (66.7)	16 (88.9)	0.2	24 (60.0)	39 (97.5)	<0.001

Badly inspired film, n (%)	15 (38.5)	16 (41.0)	0.8	23 (40.4)	23 (40.4)	>0.9	3 (17.6)	10 (58.8)	0.013	5 (55.6)	4 (44.4)	>0.9	9 (45.0)	6 (30.0)	0.3
Badly penetrated film, n (%)	33 (84.6)	36 (92.3)	0.5	38 (66.7)	54 (94.7)	<0.001	16 (94.1)	17 (100.0)	>0.9	5 (55.6)	9 (100.0)	0.082	16 (80.0)	16 (80.0)	>0.9
Miliary, n(%)	35 (89.7)	37 (94.9)	0.7	38 (66.7)	51 (89.5)	0.003	16 (94.1)	17 (100.0)	>0.9	6 (66.7)	9 (100.0)	0.2	13 (65.0)	19 (95.0)	0.044
Normal view with thymus, n (%)	5 (6.4)	41 (52.6)	<0.00 1	26 (22.8)	29 (25.4)	0.6	5 (14.7)	16 (47.1)	0.004	1 (5.6)	5 (27.8)	0.2	4 (10.0)	13 (32.5)	0.014
Normal view without thymus, n (%)	23 (59.0)	28 (71.8)	0.2	17 (29.8)	24 (42.1)	0.2	11 (64.7)	10 (58.8)	0.7	1 (11.1)	2 (22.2)	>0.9	4 (20.0)	7 (35.0)	0.3
Opacities alveolar + adenopathies, n (%)	34 (87.2)	36 (92.3)	0.7	43 (75.4)	53 (93.0)	0.010	16 (94.1)	17 (100.0)	>0.9	4 (44.4)	9 (100.0)	0.029	10 (50.0)	20 (100.0)	<0.001
Pericarditis, n(%)	31 (79.5)	35 (89.7)	0.2	42 (73.7)	54 (94.7)	0.002	14 (82.4)	16 (94.1)	0.6	9 (100.0)	8 (88.9)	>0.9	16 (80.0)	17 (85.0)	>0.9
Pleurisy, n(%)	31 (79.5)	39 (100.0)	0.005	38 (66.7)	51 (89.5)	0.003	16 (94.1)	17 (100.0)	>0.9	6 (66.7)	8 (88.9)	0.6	14 (70.0)	18 (90.0)	0.2

[1]Mc Neymar test
